# Pacing Variation in Multistage Ultramarathons: Internet-Based Cross-Sectional Study

**DOI:** 10.2196/46650

**Published:** 2023-08-23

**Authors:** Mielad Fariod, Rafael Reis Olher, Caio Victor Sousa, Volker Scheer, Ivan Cuk, Pantelis Theodoros Nikolaidis, Mabliny Thuany, Katja Weiss, Beat Knechtle

**Affiliations:** 1 Department of Orthopedic Traumatology and Reconstructive Surgery, Klinikum Frankfurt-Höchst Frankfurt Germany; 2 Department of Physical Education, University Center of Central Plateau Apparecido dos Santos Brasilia Brazil; 3 Health and Human Sciences Loyola Marymount University Los Angeles, CA United States; 4 Ultra Sports Science Foundation Pierre-Benite France; 5 Faculty of Sport and Physical Education University of Belgrade Belgrade; 6 School of Health and Caring Sciences University of West Attica Athens Greece; 7 Faculty of Sports University of Porto Porto Portugal; 8 Institute of Primary Care University Hospital Zurich Zurich Switzerland; 9 Medbase St Gallen Am Vadianplatz St Gallen Switzerland

**Keywords:** ultramarathon, pacing, gender difference, performance, variation

## Abstract

**Background:**

Ultramarathon running is the most popular ultraendurance competition in terms of the number of races and runners competing annually worldwide; however, no study has compared pacing and performance over a long period.

**Objective:**

This study analyzes the pacing of successful finishers and nonfinishers in multistage ultramarathons worldwide.

**Methods:**

A total of 4079 athletes (men=3288; women=791) competing in 99 multistage ultramarathon events from 1983 to 2021 were analyzed, including the number of participants, age, gender, rank, and running speed of successful finishers.

**Results:**

The results showed a significant increase in the number of events (n=338) and a significant increase in the number of finishers and nonfinishers (n=5575) in the ultramarathons worldwide during this period. The general linear models (GLMs) of pacing variation showed nonsignificant effects for gender (*F*_1,36.2_=2.5; *P*=.127; η_p_^2^=0.063) and age group (*F*_10,10_=0.6; *P*=.798; η_p_^2^=0.367), but it showed a significant interaction (gender × age) effect (*F*_10,2689_=2.3; *P*=.008; η_p_^2^=0.009). Post hoc analyses showed that men have a higher pacing variation than women in the under 30 years (U30), U35, U45, and U50 groups. Additionally, the fastest women’s age group (U35) had the lowest pacing variation. The GLM of pacing variation by gender and event distance showed significant effects for both gender (*F*_1,3_=18.5; *P*<.001; η_p_^2^=0.007) and distance (*F*_2,3_=20.1; *P*<.001; η_p_^2^=0.015). Post hoc analyses showed a growing pacing variation with increasing race distance for both men and women. In addition, men had a higher variation in long events. Furthermore, there was a significant main effect for both genders (*F*_1,3_=33.7; *P*<.001; η_p_^2^=0.012) and rank (*F*_1,3_=136.6; *P*<.001; η_p_^2^=0.048) on performance, with men being faster than women. Pacing varied greatly due to gender (*F*_1,3_=4.0; *P*=.047; η_p_^2^=0.001), with a lower (ie, more even) pacing variation for male athletes in the top 3 finishers. Male nonfinishers showed a higher performance than female nonfinishers (*F*_1,1340_=25.6; *P*<.001), and no difference was identified for pacing variation (*F*_1,789_=1.5; *P*=.228) based on gender. In addition, a weak but significant correlation (*r*=–0.130; *P*<.001) was identified between the average running speed and pacing variation for both female and male nonfinishers.

**Conclusions:**

In summary, multistage ultramarathon competitions showed an increasing number of competitors and a higher performance challenge. Men have a higher pacing (ie, less even) variation than women, especially observed in longer events. A higher pacing variation was associated with lower performance for men, women, and nonfinishers.

## Introduction

A multistage ultramarathon is characterized as a running race with a distance longer than the marathon distance (42,195 km) or a timed event that can vary from 6 hours to several days [1]. Consequently, 50 km is the shortest standard distance considered a multistage ultramarathon, or 6 hours for a multistage ultramarathon considering the shortest time [2]. Other ultradistance races are 50 km, 100 km, or longer for distance events, with 6 hours up to 10 days for established events [3].

In recent years, ultramarathon running has become more popular, with more organized events each year [4]. The number of runners and races in multistage ultramarathon events has climbed from 283 events in the year 1990 to over 7000 in the year 2020 around the world in the past years [5].

Several studies in the last few years have analyzed the participation and performance trends in multistage ultramarathons [1,3,6-9]. The increase in participation was partially explained by the number of women and young runners participating in these events. At the same time, the research community’s interest in understanding the factors associated with ultramarathoners’ participation, motivational and psychosocial aspects, and training characteristics [10] grew.

In endurance competitions with a known end point (with factors such as distance, time, or the principle of backward running), athletes must constantly regulate their pace. They do this to finish the race in the shortest possible time or cover the longest possible distance [11]. This process is described as pacing, which plays an important role in optimizing endurance performance that involves the capacity to cope with perceptual and physiological responses as well as race characteristics and environmental factors [12-14]. In this regard, an inappropriate pacing strategy may result in suboptimal performance, as athletes may have to deal with premature fatigue if, for instance, they choose an aggressive pacing strategy (running a faster racing pace in the beginning and continuing with a constant pace) beyond their psychophysiological capabilities [15,16].

Different exercises performed at a controlled pace, such as time-to-exhaustion tests or self-paced exercises, allow participants to pace themselves in response to their physiological and perceived exertion perturbations to maintain sustained pacing throughout the exercise [17]. This might be important in ultramarathons, as runners frequently face variable elevations (eg, sea level vs mountains) and wind conditions in a very long-running race. Multistage ultramarathons in the mountains may challenge successful pacing strategies due to variable climbs, irregular terrain, and changing winds [18].

In terms of endurance performances, different studies have investigated pacing strategies in running races with distances varying from 10 to 42 km and ranging from ~30 minutes to ~2.5 hours [19-21]. However, running races with longer distances and durations, such as ultramarathons, have been investigated less [22,23]. Furthermore, studies examining multistage ultramarathon pacing strategies have observed general positive pacing strategies in most parts of the event, which resulted in increased speed in the last 10% of the race [24-26]. Positive pacing has also been seen in shorter-distance events, like 800 m [27], 5 km [28], and 10 km [29-34]. When studying literature and other previous studies considering the pacing of men and women regarding gender differences, surprisingly, women had similar pacing as men in a half- or ultramarathon. In marathons, however, women had more even pacing than men, which confirmed the previous findings in other marathon races [35,36]. This gender difference has been correlated with differences in physiology and decision-making between women and men [34]. More studies are needed to explore this phenomenon.

Therefore, the aim of this study was to examine the number of participants worldwide in multistage ultramarathons. Several points were analyzed: first, the more extensive database, which includes the years since 1983 in the analysis; second, to look at the increasing number of participants in relation to the increasing number of events; third, to examine the performance based on age and gender; fourth, to analyze the change in performance over the last decades; and lastly, to highlight the performance gap between the genders. Based upon the findings mentioned above regarding the participation and performance trends in multistage ultramarathon races held worldwide, we hypothesized that (1) the participation worldwide increased in the last decades, (2) the participants/event ratio declined, (3) no further significant performance gap in older age groups occurred, (4) the performance among older athletes increased over the past decades, and (5) the participants were faster in most age groups, except for the youngest and oldest age groups. These assumptions could be interesting for athletes who have plans for their ultramarathon careers and for scientists who explore the athletes’ efficiency.

## Methods

### Ethics Approval

This study was approved by the institutional review board of Kanton St. Gallen, Switzerland, with a waiver of the requirement for informed consent of the participants as the study involved the analysis of publicly available data (EKSG 01/06/2010). The study was conducted in accordance with recognized ethical standards according to the Declaration of Helsinki, adopted in 1964 and revised in 2013.

### Data Selection

We included multistage ultramarathons with a minimum of 3 stages per race or event, where every lap or stage was electronically recorded. The data were extracted directly from the internet page and database of the DUV (Deutsche Ultramarathon Vereinigung e.V.) [37]. Runners who completed the full time of the race were called “Finishers” (F), and runners who dropped out of the race with partial completion were denominated as “Did Not Finish” (DNF). Both F and DNF categories of participants were considered for data analysis. The inclusion criteria for this particular study were all races with a distance greater than 42,195 km. The entire sample included data from 99 race events. From this publicly accessible results database of the DUV, we have included official results and split times for all multistage ultramarathons with more than 3 days since the first official race in 1983 to 2021. The year of the race, the race, the stage, the distance, the rank, gender, age, nationality, ranking, average speed, and the race time of each successful finisher, and DNF were recorded. Runners younger than 18 years were excluded from this study. We defined the distances as short (total distance up to 323 km), medium (total distance between 323 and 353.2 km), and long (total distance from 353.3 km and above).

### Statistical Analysis

Descriptive statistics were presented using the mean, SD, and frequencies (%). The data were tested for parametric distribution with the Shapiro-Wilk test. General linear models (GLMs) were applied to test the effect of gender (women; men), age (participants in the under 30 years [U30], U35, U40, U45, U50, U55, U60, U65, U70, U75, and U80 groups), ranking (top 3 finishers vs others), and interaction (gender × age; gender × ranking; age × ranking) on dependent variables (ie, average speed and pacing variance). Pacing variance is represented by the coefficient of variation of the mean pace across each stage of a multistage event [38]. The pacing variance was calculated individually. The least significant technique was used as a post hoc test for pairwise comparisons. We applied the Pearson correlation coefficient [39] to identify any potential correlation between pacing variation and performance in different conditions. Athletes who dropped out of the race were classified as DNF and analyzed separately from those who finished. Eta squared (ŋ^2^) was calculated for the ANOVAs where the effect sizes of 0.01, 0.06, and above 0.14 were considered small, medium, and large, respectively [40]. The level of statistical significance was set at *P*<.05. All statistical analyses were carried out using SPSS (version 28; IBM Corp).

## Results

**Figure 1:**
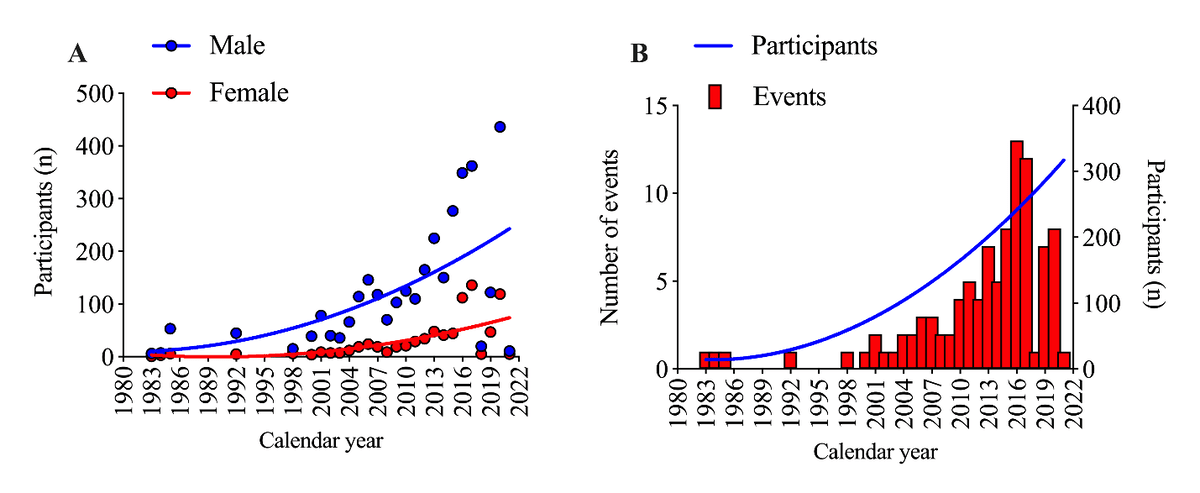
The number of participants and the number of events throughout the calendar years.

The analyses included a total of 4079 athletes (men=3288; women=791) competing in 99 multistage ultramarathon events from 1983 to 2021. The number of participants gradually increased and climbed yearly for both men and women until 2020, but men were the majority yearly. Similarly, the number of events grew throughout the years (Figure 1).

Approximately one-third of the participants (men=32.2%, 1060/3288; women=35.9%, 284/791 dropped out of the race and were classified as DNF (Table 1). Events with fewer stages (shorter) had a higher dropout rate than medium or longer events (n=1111, 51% vs n=220, 33%). The rate of DNF across age groups ranged from 28% to 50%.

**Figure 2:**
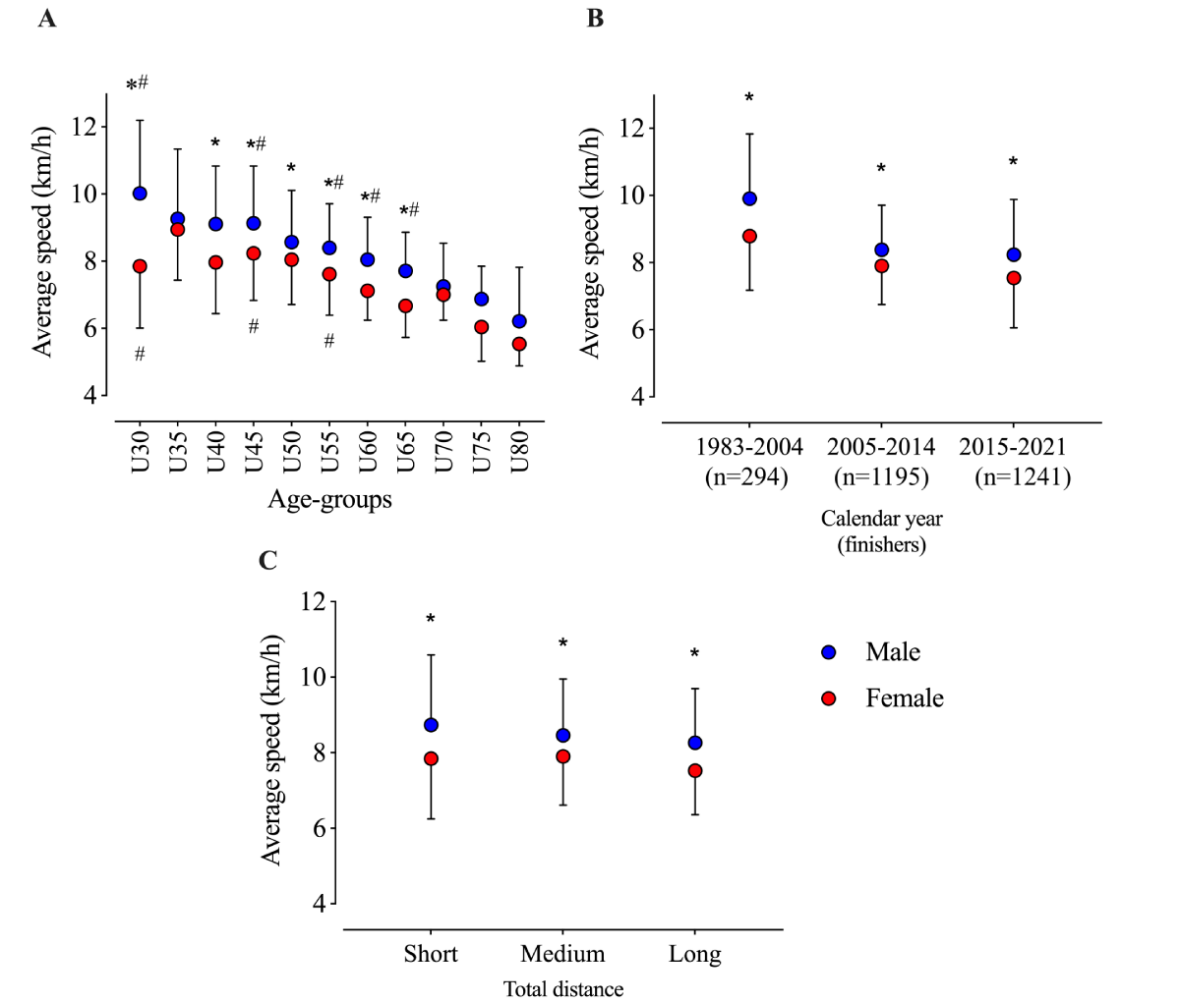
Performance in multistage marathons by age group, calendar years, and total distance. *Significant difference between men and women; #Significant difference versus the next age group.

The first GLM of average performance showed significant effects for gender (*F*_1,41.7_=30.8; *P*<.001; η_p_^2^=0.425), age group (*F*_10,10_=11.4; *P*<.001; η_p_^2^=0.921), and interaction (*F*_10,2689_=2.1; *P*=.02; η_p_^2^=0.008). Post hoc analyses showed that men were faster than women in all age groups except U35, U70, U75, and U80. In addition, the fastest men’s age group was U30, whereas the fastest women’s age group was U35 (Figure 2). Men were also faster than women across all decades (Figure 2) and different race distances (Figure 2).

**Table 1:** Number of participants who finished and those who did not finish the multistage marathon.

			Finishers, n (%)		DNF^a^, n (%)
**Gender**					
	Men	2228 (67.8)		1060 (32.2)	
	Women	507 (64.1)		284 (35.9)	
**Distance**					
	Short^b^	1069 (49.0)		1111 (51.0)	
	Medium^c^	762 (98.3)		13 (1.7)	
	Long^d^	903 (67.0)		220 (33.0)	
**Performance**					
	Slow	910 (58.2)		653 (41.8)	
	Medium	910 (68.9)		411 (31.1)	
	Fast	912 (76.6)		278 (23.4)	
**Age group**					
	U30^e^	74 (59.2)		51 (40.8)	
	U35	137 (66.5)		69 (33.5)	
	U40	272 (71.8)		107 (28.2)	
	U45	418 (69.0)		188 (31.0)	
	U50	517 (66.7)		258 (33.3)	
	U55	527 (67.0)		260 (33.0)	
	U60	349 (65.6)		183 (34.4)	
	U65	260 (72.0)		101 (28.0)	
	U70	118 (72.0)		46 (28.0)	
	U75	35 (67.3)		17 (32.7)	
	U80	9 (50.0)		9 (50.0)	

^a^DNF: did not finish.

^b^Total distance up to 323 km.

^c^Total distance between 323 and 353.2 km.

^d^Total distance from 353.3 km and above.

^e^U30: age group of under 30 years.

**Figure 3:**
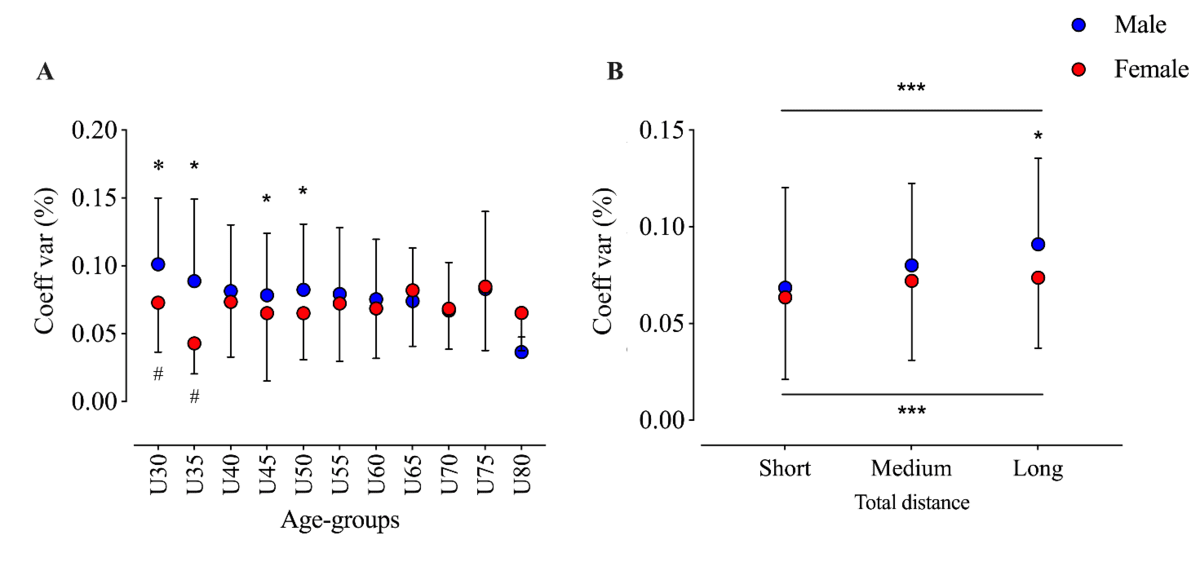
Pacing variation in multistage marathons by age group and total distance. *Significant difference between men and women; #Significant difference versus the next age group; ***All groups are significantly different from each other. Coeff: coefficient; var: variation.

The GLM of pacing variation showed nonsignificant effects for gender (*F*_1,36.2_=2.5; *P*=.13; η_p_^2^=0.063) and age group (*F*_10,10_=0.6; *P*=.80; η_p_^2^=0.367), but it showed a significant interaction (gender × age) effect (*F*_10,2686_=2.3; *P*=.008; η_p_^2^=0.009). Post hoc analyses showed that men have a higher pacing variation than women in U30, U35, U45, and U50. Additionally, the fastest women’s age group (U35) had the lowest pacing variation (Figure 3). The GLM of pacing variation by gender and event distance showed significant effects for both gender (*F*_1,3_=18.5; *P*<.001; η_p_^2^=0.007) and distance (*F*_2,3_=20.1; *P*<.001; η_p_^2^=0.015). Post hoc analyses showed a growing pacing variation with increasing race distance for both men and women. In addition, men had a higher variation in long events (Figure 3).

**Figure 4:**
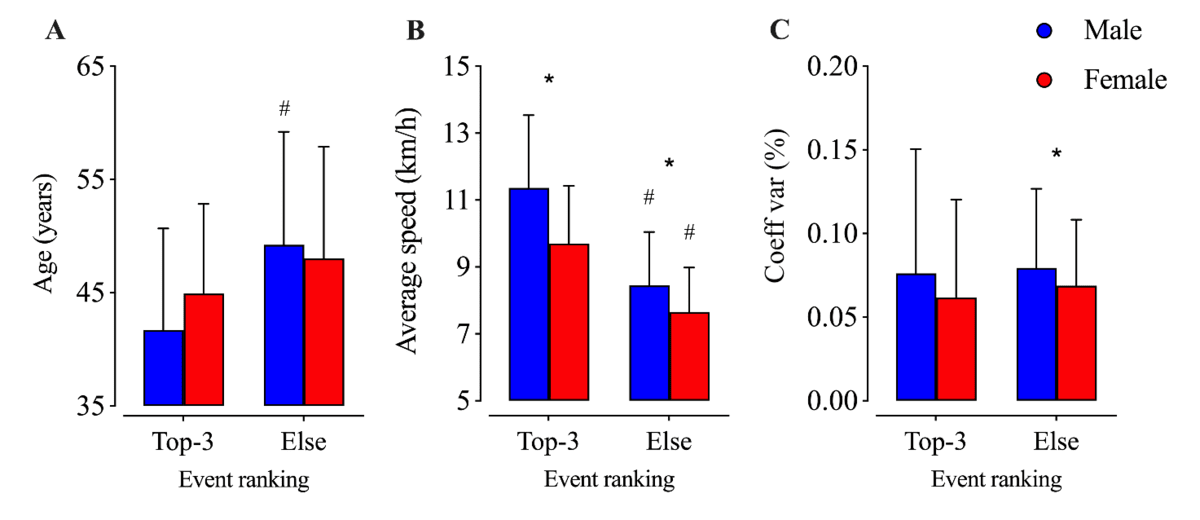
Age, performance, and pacing variation by rank and gender. *Significant difference between men and women; #Significant difference between rank status. Coeff: coefficient; var: variation.

When divided by rank (top 3 finishers vs others), a significant rank effect was found with age as a dependent variable (*F*_1,3_=15.9; *P*<.001; η_p_^2^=0.006), with the top 3 male athletes being younger than their gender-matched peers (Figure 4). The average performance showed a significant effect for gender (*F*_1,3_=33.7; *P*<.001; η_p_^2^=0.012) and rank (*F*_1,3_=136.6; *P*<.001; η_p_^2^=0.048), with men being faster than women in both scenarios and, as expected, the top 3 athletes being faster than other athletes for both men and women (Figure 4). Pacing variation showed a significant effect for gender (*F*_1,3_=4.0; *P*=.047; η_p_^2^=0.001), with a higher pacing variation for men in athletes other than the top 3 (Figure 4).

**Figure 5:**
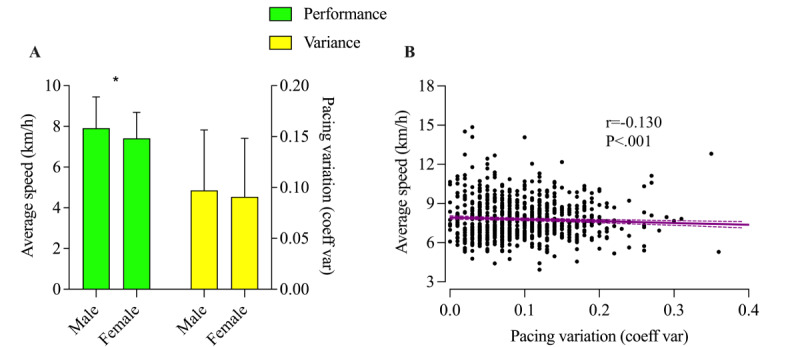
Performance and pacing variation by gender of DNF athletes. *Significant difference between men and women. Coeff: coefficient; var: variation.

Male nonfinishers showed a higher performance than female nonfinishers (*F*_1,1340_=25.6; *P*<.001; η_p_^2^=0.002), and no difference was identified for pacing variation (*F*_1,789_=1.5; *P*=.23; η_p_^2^=0.048). In addition, a trivial but significant correlation (*r*=–0.130; *P*<.001) was identified between average running speed and pacing variation for both female and male nonfinishers (Figure 5).

**Figure 6:**
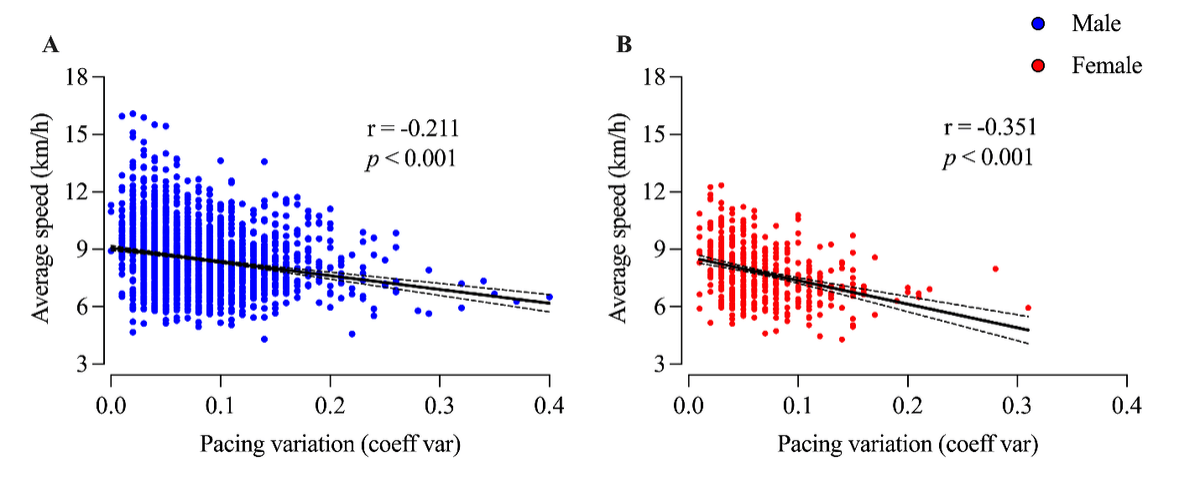
Correlation between pacing variation and average running speed. Coeff: coefficient; var: variation.

Negative and significant correlations were identified between performance and pacing variation for both men (*r*=–0.211; *P*<.001) and women (*r*=–0.351; *P*<.001; Figure 6). An increase in pacing variation is related to the performance decline.

## Discussion

### Principal Findings

This study examined the participation and performance in multistage ultramarathon events worldwide over 38 years. Our aim was to test 4 hypotheses. Our initial hypotheses were as follows: first, the number of participants have increased in the last several decades; second, the participants or event ratio have declined; third, there has been a significant performance gap between different age groups; and fourth, there is a difference in pacing variations in the top 3 athletes between men and women and between finishers and nonfinishers. Our findings show that: first, the number of events and participants increased, but men still represented the majority of participants; second, the performance gap between men and women remained significant; third, the pacing variation in the top 3 did not differ between men and women, but men had a higher pacing variation than women; fourth, although the pacing variation in nonfinishers did not differ between men and women, a higher pacing variation was associated with lower performance for men, women, and nonfinishers.

The first important finding where we could confirm our hypothesis is that the number of participants gradually increased yearly for both men and women until 2020, with men being the majority each year. Similarly, the number of events in correlation to the number of participants has grown throughout the years [37]. Reaching the plateau in 2015 and decreasing during the COVID-19 pandemic years in 2019-2021 [41], men remained the most represented compared to women.

Similar ﬁndings with an increase in participants were reported for the worldwide fastest runners in 100-km ultramarathons [32]. An additional study investigating the participation trends in multistage ultramarathon races also dispatched an increase in the number of ﬁnishers during the last 3 decades [42]. The number of participants in other ultramarathons also increased, for example, when evaluating worldwide data from 24-hour ultramarathons [43] and worldwide data from 6-72–hour, 6-day, and 10-day ultramarathons [9].

The increase in participants in this study was mostly related to the higher number of male participants. Nevertheless, in our study, the number of female participants has grown in the last decade, from 2010 to 2020, with men remaining the majority. A previously conducted study of multistage ultramarathons worldwide between 2000 and 2010 presented similar results: the number of participants increased exponentially for both genders, but the minority of women (15%) participants remained the same [8]. This might be explained by the higher popularity of multistage ultramarathons in the last decade.

The second important finding was that a significant performance gap was found between men and women, especially in older age groups, in terms of age, year, and distance. This confirms our hypothesis. Analyses showed that men are faster than women in all age groups except U35, U70, U75, and U80. In addition, the fastest men’s age group is U30, whereas the fastest women’s age group is U35. Men were also faster than women across all decades. This was especially observable in groups U30, U40, and U60 and different races with short, medium, and long distances.

Conversely, athletes running shorter distances, like in the “New York City Marathon,” revealed a significant decline in performance among elite marathon runners aged 36 years and older [44]. Similarly, the analysis of several marathon races showed a significant performance gap between the age groups of athletes over 56 years and recreational runners [45]. A significant performance gap was also observed from age 50 onward in mountain running [46]. However, in our study, with extensive data collection for ultramarathon events, performance differences could be found at all ages and after 60 years.

Furthermore, it has been observed that lifelong endurance training can function and maintain muscle mass in the elderly [47]. Similarly, aerobic fitness per unit of body mass could be a factor in endurance performance, which showed a correlation with the level of competition in ultra–long-distance runners [48]. The second ventilation threshold was also the best aerobic fitness variable [44]. These physiological variables, like aerobic fitness per body unit, muscle mass, or the second ventilation threshold, could also be used for further studies to investigate endurance performance in older athletes.

Half-marathon and ultramarathon runners adopted variable pacing instead of maintaining constant speed throughout the race [49]. Variable self-pacing has been shown to present certain advantages, that is, enhancement of critical power and high-intensity exercise performance compared to constant work rate cycling exercise [50]. In addition, the rate of perceived exertion has been shown to be associated with pacing [51] and might vary across races [52]. Moreover, compared to running alone, it was found that head-to-head competition performance improved [53]. Although this aspect was not examined in this study, it can be assumed that head-to-head competition exerted a similar influence on both race distances since the marathon and ultramarathon races were massive events. Overall, pacing should be considered a complex system where an individual’s responses interact with the environment [54]. In this context, athletes were requested to balance behavior and thinking (self-regulation) to optimize their speed across the race [55,56]. Future studies should verify this aspect too.

The third important finding was the pacing difference between the top 3 athletes. We could confirm our hypothesis regarding a significant rank effect found with age as a dependent variable, with the top 3 male athletes being younger than their gender-matched peers. Pacing variation showed a significant effect on gender, with higher pacing variation for men in athletes other than the top 3.

Furthermore, there were more significant performance gaps between male and female participants. Similar or other results can be shown in shorter 80-km ultramarathons, where the performance gap decreased in the early decades [45]. In an analysis of ultramarathons of varying lengths, women were also able to reduce the performance gap to men in 6-, 72-, 144-, and 240 hour races since 1975 [57], in 24 hour races since 1998 [11], and throughout all the decades where 50-km ultramarathons worldwide were held [36].

In the last decade, the top 3 male athletes were not much faster than the top 3 female athletes. The performance of both genders increased in the last period. However, the performance gap stays constant every decade for both genders. The top 3 male athletes remained significantly faster than the top 3 female athletes. Meanwhile, a worldwide study of the fastest women and men 100-km ultramarathoners shows a decline in the performance gap. In this study, it was observed that the performance gap decreased from 56% in 1965 to 16% in 2012 [7]. Additionally, the performance gap of the top 10 runners decreased from 46% in 1975 to 14% in 2012 in 100-km ultramarathons worldwide [7] and 24 hour ultramarathons worldwide [58].

A fourth and last important finding was that male nonfinishers performed better than female nonfinishers, and no difference was observed for pacing variation. In addition, a weak but significant correlation was identified between average speed and pacing variation for all nonfinishers. Negative and significant correlations were identified between performance and pacing variation for both men and women.

The findings mentioned above might be explained by the fact that, with the increasing duration of a race, the fatigue of the athletes increases as well. It is easier to maintain a steady and fast speed during the 6 hours in 1 or more stages or during a 24 hour race. Other studies have shown that the relative running speed of the athletes decreases with the duration of an ultraendurance event. In addition, nutritional factors such as dehydration and gastrointestinal problems contribute to dropouts in ultraendurance races [59].

A further observation was that athletes in the ultramarathon races demonstrated more variable pacing than those in the 6 hour and 12 hour races. As we already showed, the finishers in the 6 hour races were the ones with the fastest race speed [60]. The expectation was to see less variable pacing, as others have already reported that faster finishers showed fewer variations in running [20]. This finding is in accordance with the literature. It has already been observed that half-marathon men and women runners keep a more even pace than marathon or ultramarathon men and women runners [51]. This trend can also be observed in the nonfinisher group, which showed no significant correlation in pacing variation between men and women. The pacing is more consistent and easier to reach in shorter events than in longer ultramarathon events. The changes in running speed may be a consequence of fatigue during a prolonged exercise competition. Physical exhaustion naturally plays a larger role in longer-running events, resulting in more variable pacing in ultramarathon races than in shorter races [61].

Our data showed that a higher pacing variation was associated with lower performance for men, women, and nonfinishers, in contrast with the published data [62], wherein the fastest times were associated with fewer changes in running speed and performance during a race [38]. The negative relationship between performance and pacing variation in our study [63], who observed a very strong negative correlation between performance and pacing variation in a 75-km mountain race, and [64], who showed a more even pacing in the fastest finishers of 161-km and 101-km races [61]. A high pacing variation might result from early effort perception and to incorrect use of the physiological potential for nonelite runners [65]. Furthermore, it has been suggested that optimal pacing might depend on several factors, including the kind of locomotion, the round of competition, and the criteria for competitive success [66].

This study is not free of limitations. For instance, future studies should include more participant characteristics such as aerobic fitness per unit of body, muscle mass, fat mass, or years of training since these variables were previously related to ultramarathon performance and could also be related to pacing variation [67]. Despite the large amount of data available on the DUV website, these characteristics cannot be retrospectively recorded in our study. Therefore, the gap in this study can only be closed by using a different study design to identify the origin of the data observed. However, a weakness of this study is the low number of participants in the higher age groups despite the high volume of data. A trend between age and performance was found, but this result could be due to the overestimation of higher age groups. The importance of making decisions about optimal energy use during a race has been highlighted previously [68] and suggested that how an athlete manages her effort during an exercise task may impact performance [69]. Thus, the findings have important practical applications for participants in ultramarathon races, who are advised to follow a relatively even pace and start the race at a running speed that they may relatively maintain throughout the terrain.

### Conclusions

In conclusion, this study shows an increasing number of events and participants, but men are still a significant majority compared to women. Men also show a higher pacing variation than women, especially in longer events. Furthermore, higher-ranked athletes are mostly younger men than women. Pacing variations in the top 3 runners of all events between 1983 and 2021 do not differ between men and women. In addition, higher pacing variation is associated with lower performance for men, women, and DNF athletes. Further analyses of the growing age group of older participants could provide information on performance development and gender differences in old age. For coaches and professional athletes, this study shows that despite increasing age, an increase in performance can still be achieved in the next few decades and that the number of participants among older athletes will increase in the future. Differences in terms of pacing and performance between men and women athletes should also be taken into consideration. Coaches could use these findings to improve the performance of novice runners. For example, these novice runners could be advised by coaches to pay attention to optimizing their pacing strategies and running speed (ie, overexertion in the first half, two-thirds, and so on, of a race leads to variable pacing in the remainder of the race and can therefore greatly hinder performance times). According to this, there is still much potential in older athletes’ training economies and training volumes.

## Abbreviations

DNF: Did Not Finish
DUV: Deutsche Ultramarathon Vereinigung e.V.
F: Finishers
GLM: general linear model

## Data Availability

For this study, we have included official results and split times from the website of the DUV.
